# Roles and Applications of Red Blood Cell-Derived Extracellular Vesicles in Health and Diseases

**DOI:** 10.3390/ijms23115927

**Published:** 2022-05-25

**Authors:** Lan Yang, Shiqi Huang, Zhirong Zhang, Zhenmi Liu, Ling Zhang

**Affiliations:** 1 Key Laboratory of Drug-Targeting and Drug Delivery System of the Education Ministry, West China School of Pharmacy, Sichuan University, Chengdu 610041, China; yanglanlancc@163.com (L.Y.); seikil7@sina.com (S.H.); zrzzl@vip.sina.com (Z.Z.); 2 Med-X Center for Materials, West China School of Public Health, Sichuan University, Chengdu 610041, China; zhenmiliu@scu.edu.cn; 3 Med-X Center for Materials, College of Polymer Science and Engineering, Sichuan University, Chengdu 610065, China

**Keywords:** red blood cells, extracellular vesicles, targeting delivery, diagnosis

## Abstract

Red blood cell-derived extracellular vesicles (RBCEVs) are vesicles naturally produced by red blood cells and play multiple roles such as acting as cell-to-cell communication messengers in both normal physiological and diseased states. RBCEVs are highly promising delivery vehicles for therapeutic agents such as biomolecules and nucleic acids as they are easy to source, safe, and versatile. RBCEVs autonomously target the liver and pass the blood–brain barrier into the brain, which is highly valuable for the treatment of liver and brain diseases. RBCEVs can be modified by various functional units, including various functional molecules and nanoparticles, to improve their active targeting capabilities for tumors or other sites. Moreover, the RBCEV level is significantly shifted in many diseased states; hence, they can also serve as important biomarkers for disease diagnoses. It is clear that RBCEVs have considerable potential in multiple medical applications. In this review, we briefly introduce the biological roles of RBCEVs, presented interesting advances in RBCEV applications, and discuss several challenges that need to be addressed for their clinical translation.

## 1. Introduction

Cell-to-cell communications are mediated not only by secreted/leaked molecules such as cytokines, but also via released extracellular vesicles (EVs) and nanotubes [1,2], where many EVs are involved in the exchange of proteins, nucleic acids, and lipids [3,4]. Growing evidence indicates that EVs play major roles in numerous physiological and pathological processes, including aging, cancer, infectious diseases, and obesity [5,6]. Due to their potential in medical applications, a large number of studies on EVs have been recently published.

**Figure 1:**
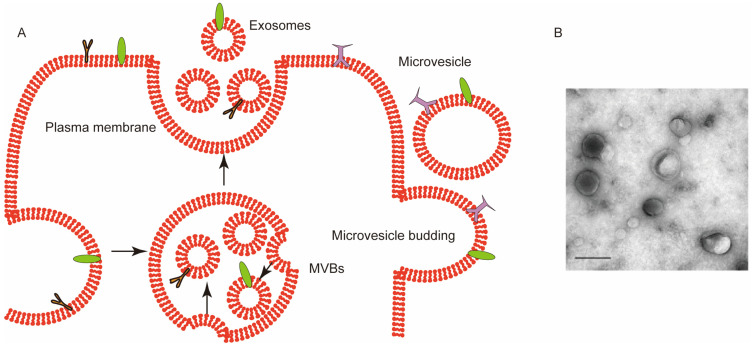
(**A**) Biogenesis of extracellular vesicles (EVs). EVs used in medical research are mostly classified into exosomes and microvesicles. The fusion of multivesicular bodies (MVBs) with the plasma membrane is followed by the invagination of the membrane to form MVBs to generate exosomes whereas microvesicles are produced by outward budding. The arrows represent EVs formation process. (**B**) Transmission electron microscope (TEM) image of RBCEVs. Scale bar: 100 nm. Adapted from [11].

Currently, it is widely accepted that EV is an umbrella term describing heterogenous lipid membrane-limited vesicles naturally produced by cells. There are three major types of EVs: exosomes; microvesicles; and apoptotic bodies. These three types of EVs have distinct biological origins and show different properties such as composition, contents, and physical appearances. Exosomes originate from the invagination of the plasma membrane to form endosomes, which can subsequently mature into multivesicular bodies (MVBs) and finally release their vesicular contents after a fusion with the cell membrane [7]. Thus, exosomes are ultimately derived from the plasma membrane, which experiences multiple maturation steps. Exosomes have a relatively narrow size range, usually between 50 and 150 nm. Microvesicles, on the other hand, cover a wide range between 50 nm and 1000 nm. These vesicles are generated by direct outward budding and the scission of the plasma membrane; however, the detailed mechanism has not been fully uncovered [8]. It is suggested that microvesicles have a greater heterogeneity than exosomes, further complicating the research of these vesicles. In comparison with these two types of EVs, apoptotic bodies are not normally produced by healthy cells, but by apoptotic cells during a process named apoptotic cell disassembly. These EVs can contain highly different substances from the dying cell, with a size range of 1–5 µm [9]. It has been found that the composition and properties of exosomes and microvesicles are generally easier to control and work with than apoptotic bodies. There are normally a greater number of cells in conditions that produce exosomes and microvesicles both in vivo and in vitro; hence, these EVs are more commonly used in medical research (Figure 1A) [10].

There are remaining controversies, which usually stem from the technical challenges of isolating and analyzing EVs. Common methods applied to EV purification such as ultra-centrifugation and immunoprecipitation usually result in a mixture of different types of EVs [12]. Worse still, cells also produce other nanosized particles in addition to EVs such as exomeres [13]. These non-EV particles can also remain with an isolated EV and further complicate the issue. Therefore, EV research usually uses samples that are not strictly “pure”, and the description of EVs is, therefore, based on mixed subpopulations of particles. This issue is more profound in a clinical translation as the large-scale production of EVs or the examination of patient samples likely uses EVs that are even less “pure”. It might be impossible to acquire a single type of EV in practical settings. In this way, in many cases (especially in clinical applications), it is acceptable or even beneficial to consider less pure EVs instead of pursuing strictly defined exosomes or microvesicles. Thus, in the following text, we do not strictly differ between the different types of EVs unless the difference brings a significant impact on the specified properties or usages.

Red blood cells (RBCs) have a distinct biconcave round shape and are the most abundant type of blood cells in the blood [14]. Their main functions are to transport oxygen and carbon dioxide as well as release adenosine triphosphate (ATP) and nitric oxide (NO) [15]. RBC-derived extracellular vesicles (RBCEVs) also include different subpopulations such as exosomes and microvesicles. For example, vesicles may form during the normal aging of circulating erythrocytes due to complement-mediated calcium influx, plasma membrane budding, and subsequent vesicle shedding [16]. As with other EVs, although RBCEVs are derived from RBCs, their membrane compositions and internal contents are not the same. During the formation of RBCEVs, cytoskeletal components are partially absent or even completely absent whereas glycosylphosphatidylinositol (GPI) connexins and lipid raft markers such as ganglioside M1 or stomata are relatively enriched [17]. The RBCEVs in this study were generally visualized as round vesicles and ~140 nm in size by a transmission electron microscope (Figure 1B).

Proteomic analyses and transcriptomic studies have recently been applied to the study of RBCEVs [18]. The key data such as protein and nucleic acid composition are commonly stored in Vesiclepedia, Evpedia, and other databases [19,20,21]. RBCEVs are mainly composed of lipids, proteins, and nucleic acids (miRNAs). These lipids are mainly cholesterol, phosphatidylcholine (PC), phosphatidylethanolamine (PE), and phosphatidylserine (PS) [22]. Hemoglobin, anion transporters, glycoproteins, multivesicular body fusion proteins, membrane-associated proteins, carbonic anhydrase, and other enzymes are also found in/on RBCEVs [23].

Arguably, RBCs are an excellent source for the production of EVs for drug delivery. RBCEVs have several unique advantages compared with other types of cell-derived EVs. First, RBCEVs lack both nuclear and mitochondrial DNA, which is advantageous for delivering genetic materials [24]. Moreover, RBCEVs produced by O blood type cells may be used in allogenic individuals and the blood cells are easy to obtain from donors. This mitigates two major issues of the large-scale production and applications of EVs, and may also reduce the production cost [25]. Furthermore, RBCEVs can escape macrophage clearance through the binding of CD47 to inhibitory receptor signal regulatory protein α [26]. Generally, RBCEVs exhibit an excellent clinical application potential as drug delivery vehicles as well as biomarkers in disease diagnoses.

It is clear that RBCEVs play important roles in the human body and have unique advantages in medical applications. This review introduces selected studies, emphasizes their major roles in healthy and diseased states, and presents recent progresses as well as possible advances in RBCEV-based carriers in tissue-targeting areas for other interesting medical uses, helping to build a basic idea of this field and providing a starting point to gather further possibilities.

## 2. Roles of RBCEVs in Healthy and Diseased States

As natural products of RBCs, RBCEVs are loaded with proteins, lipids, and miRNAs. They circulate in the blood and can interact with numerous tissues/cells to influence their functions, thus playing roles in physiological and pathological conditions. If the RBCEV level in healthy states is assumed to be normal, then this level is generally elevated in aging and diseased states or under other stressed conditions.

### 2.1. In a Healthy State

The first discovered function of RBCEVs was that they remove excess proteins and membranes from RBCs such as transferrin receptors, acetylcholinesterase, and hemoglobin [27,28]. RBCEVs can protect RBCs, clearing dangerous molecules and preventing their early clearance from circulation [29,30]. In addition, RBCEVs partly inherit the role of RBCs. RBCEVs are critical for communicating with endothelial cells to regulate NO and O_2_ homeostasis [25,31]. Under storage conditions, RBCEVs react faster with NO than intact red blood cells, causing strong vasoconstriction [25]. RBCEVs can affect a variety of immune cells [32,33,34]. RBCEVs can promote the production of proinflammatory cytokines (interleukin (IL) 2, 7, and 15) and tumor necrosis factor alpha (TNFα) by interacting with macrophages [34]. RBCEVs also increased a proliferation in CD4^+^ and CD8^+^ T cells by influencing the antigen-presenting cells [32]. Currently, our understanding of RBCEVs is still at an initial stage and plenty more is yet to be discovered. 

### 2.2. In Diseased States

#### 2.2.1. RBCEVs and Heart Disease

There is increasing evidence that the plasma concentrations of RBCEVs are elevated in the development of cardiovascular-related disease [35]. RBCEVs play an important role in the pathogenesis and progression of cardiovascular and related metabolic diseases. On one hand, RBCEVs enhance tumor necrosis factor alpha (TNF-a) production in monocytes, increase mitogen-induced CD4^+^ and CD8^+^ T cell proliferation, and induce endothelial cell apoptosis, leading to vascular dysfunctions [22,24]. On the other hand, RBCEVs may also play a cardioprotective role in various cardiac diseases such as ischemia reperfusions [25,36]. In the treatment of acute myocardial infarctions, in order to protect the heart, a distal ischemia modulation (Rcond) of the organ or tissue is induced by a short period of ischemia and reperfusion prior to the myocardial reperfusion. Rcond induces human endothelial-derived and procoagulant RBCEV release, which may protect the heart [36]. Therefore, it appears that RBCEVs may be a double-edged sword in cardiovascular disease as they both promote the disease process and play a role in protecting cardiomyocytes [25,26]. The underlying mechanisms of cardiometabolic diseases are complicated [37]. Studying the molecular mechanisms of the formation and release of RBCEVs, as well as their specific functions in intercellular communication, may provide new perspectives and therapeutic strategies for improving the prognosis of cardiometabolic diseases.

#### 2.2.2. RBCEVs and Parkinson’s Disease

An immune system dysfunction, including elevated levels of peripheral blood mononuclear cells and inflammatory cytokines, is an important feature of the pathogenesis of Parkinson’s disease (PD) [38]. An abnormal accumulation of α-synuclein (α-syn) is a major component of PD pathology [39], with lysosomes playing a key role in α-syn degradation [40]. Different studies [41,42] have found that a lysosomal glucocerebrosidase Gcase deficiency leads to the accumulation of α-syn; meanwhile, an increase in α-syn may inhibit the function of Gcase, thereby inducing a bidirectional pathogenic cycle. Compared with healthy subjects, the RBCEVs of PD patients contain more α-syn [43]. Pathological oligomeric α-synuclein carried by the RBCEVs of PD patients plays a central role in overactivated immunity in the circulating monocytes through receptor-mediated endocytosis and LRRK2 activation [43]. Thus, RBCEVs may have potential in the early diagnosis and treatment of PD. Nevertheless, the exact mechanistic links between these changes and PD progression is still unclear and more work is needed.

#### 2.2.3. RBCEVs and Malaria

Malaria is caused by an infection of *Plasmodium falciparum*, which spends considerable time inside the RBCs. RBCs infected by *P. falciparum* are able to transfer DNA (drug resistance and fluorescent proteins genes, etc.) through the RBCEVs, helping the parasite to survive times of stress [44]. Importantly, these EVs shed by *P. falciparum*-infected erythrocytes allow the parasite to transmit, receive, and disseminate messages favoring population growth under stress and non-stress conditions [44]. Therefore, cell-to-cell communication facilitates parasite differentiation and activation, enabling it to transmit to mosquito vectors [44]. Recent observations have shown that severe malaria is associated with a dramatic increase in EVs in the plasma of malaria patients [45]. It has also been demonstrated that the release of RBCEVs contributes to the local and systemic production of proinflammatory cytokines and chemokines, leading to vascular dysfunctions and promoting endothelial cell activation [46]. In cerebral malaria, the disruption of the blood–brain barrier can promote substances such as RBCEVs to enter the brain from the periphery and target cells such as microglia, which may contribute to the enhancement of inflammation in cerebral malaria [47]. It is likely that other RBC-related diseases may also have a close link to RBCEVs.

#### 2.2.4. RBCEVs and β-Thalassemia

Beta-thalassemia (β-TM) is a chronically challenging disease with ineffective erythropoiesis as a major pathophysiological factor [48]. Although the treatment outcomes for β-TM patients have improved over the past few years, patients still suffer from many of the complications associated with this disease such as a high incidence of thromboembolic events [49]. One study [50] has shown that the analysis of RBCEVs in β-TM patients can reflect the spleen status, hypercoagulability, and ineffective erythropoiesis as well as serving as biomarkers of the disease dynamics, supporting the prediction of complication risks and optimal treatments.

#### 2.2.5. RBCEVs and Systemic Erythematous Lupus

RBCEVs in disease states often have proinflammatory and procoagulant effects. Patients with systemic erythematous lupus (SLE) exhibit high levels of phosphatidylserine-positive (PS+) RBCEVs. Past studies have shown that elevated levels of the circulating procoagulant RBCEV leads to increased thrombotic and hypercoagulable states in sudden nocturnal hemoglobinuria (PNH) and hemolytic disease [51,52]. SLE patients are no exception, and thrombotic events are positively associated with high levels of PS+ RBCEVs [53]. These all demonstrate the relevance of RBCEVs to thrombosis, and studies have shown that RBCEVs not only coagulate by exposure to PS, but also initiate thrombin generation in an FXII-dependent manner independent of the tissue factor (TF) [54]. Therefore, investigating the mechanisms of RBCEV biogenesis and PS exposure has a therapeutic value for RBCEV-induced thromboses.

#### 2.2.6. RBCEVs and Other Diseases

**Figure 2:**
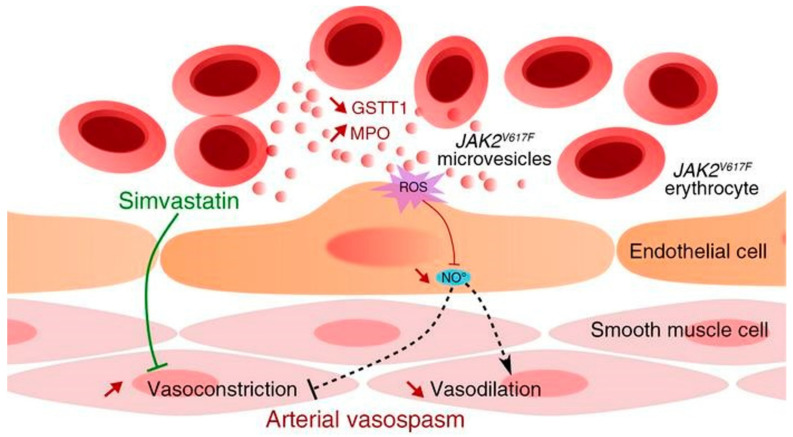
RBCEVs are associated with increased arterial constriction in Bcr/Abl-negative myeloproliferative neoplasms (MPNs) as RBCEVs carry MPO, which provides a prooxidant phenotype in endothelial cells, resulting in an increased arterial constriction. Reprinted with permission from [56]. Copyright 2020, American Society For Clinical Investigation.

RBCEVs can interfere with NO homeostasis by increasing ROS production, leading to an endothelial dysfunction [55,56]. RBCs and RBCEVs normally maintain a pro- and antioxidant balance in the blood circulation. However, RBCEVs shed by RBCs under storage conditions or during disease activate neutrophils characterized by a rapid release of reactive oxygen species. It has been reported that increased oxidative stress in endothelial cells caused by circulating RBCEVs exacerbates the vasoconstrictive response, which is an important cause of death in patients with *JAK2^V617F^* myeloproliferative neoplasms (MPNs) [56] (Figure 2). The activation of neutrophils by RBCEVs may link to the specific accumulation of lysophospholipids, which may cause transfusion-related acute lung injuries [17].

RBCEVs are also often linked with inflammation. In the inflammatory fluid of patients with arthritis, RBCEVs were found to be involved in the inflammatory process as a source of the lipid mediator LPA [22]. RBCEVs are critical for the dysregulation of hemostasis and exhibit associated procoagulant effects in several diseased states [54]. Exosomes from human cord blood plasma play an important role in wound healing by promoting angiogenesis and the differentiation of M2 macrophages, thereby facilitating the transition from inflammation to proliferation [57]. 

In summary, these studies suggest that RBCEVs serve as vehicles for delivering messages to tissues and organs, possibly at remote sites. This indicates that RBCEVs are well-positioned to transmit normal or abnormal information around the whole body, possibly playing systemic roles [58,59]. So far, it seems that RBCEVs level increase and drive disease progression in most reported diseased states. This implies that reducing the level of RBCEVs or blocking their messenger function may assist in the treatment of these diseases.

## 3. Applications

### 3.1. RBCEVs Serve as Target Delivery Vectors

As introduced, RBCEVs are naturally occurring secretory membrane vesicles that are basically ubiquitously distributed; hence, they are mostly non-toxic and well-tolerated in vivo. They are natural carriers of many small molecules as well as macromolecules; hence, they can be easily adopted for carrying various drugs and agents. It has been discovered that RBCEVs may pass a few important barriers in vivo (e.g., the blood–brain barrier) and may be able to home in on certain tissues. Furthermore, the membrane composition of RBCEVs enables them to directly fuse with the cell membrane and then transfer the loaded cargos to the target cell [60]. Therefore, engineered RBCEVs may simultaneously achieve multiple valuable functions for delivery systems such as immune evasion, specific cellular internalization, a long circulating half-life, and minimal toxicity. These provide several advantages compared with various other nanodelivery platforms such as widely used synthetic liposomes and lipid-based nanoparticles [61].

RBCEVs also have unique advantages in carrying various cargos. First, they can efficiently encapsulate and protect chemical drugs. With a lipid bilayer membrane structure, their lumen can store water-soluble drugs; hydrophobic drugs can be received by the hydrophobic region of the lipid bilayer whilst protecting the contents from the environment for an extended time length. By efficiently entrapping the hydrophobic drug camptothecin (CPT) into RBCEVs, it was found that the vesicles did not induce hemolysis [62]. A long-lasting sustained release was also observed. Other nanoparticles smaller than EVs such as ultra-small superparamagnetic iron oxide particles could also be efficiently encapsulated by RBCEVs. These particles were then delivered into mesenchymal stem cells (MSCs), forcing the labeled MSCs to undergo MRI imaging [63], which allowed the transplanted stem cells to be tracked in the body. RBCEVs are, potentially, good carriers of nucleic acids for gene therapy. So far, ~70% of gene therapy clinical trials have used virus-based carriers such as retroviruses, lentiviruses, adenoviruses, and adeno-associated viruses (AAVs) to deliver nucleic acids [64]. Although theses vectors enjoyed success in many cases, the viral vectors still suffered from several limitations, including possible carcinogenicity, non-ignorable immunogenicity, a limited DNA packaging capacity, and difficulties in vector production [65]. RBCEVs, in contrast, performed well in these aspects. For example, RNA strands were efficiently capsuled in RBCEVs and were delivered to leukemia and breast cancer cells efficiently and without any cytotoxicity [11]. Plasma exosomes (exosomes from peripheral blood, which are mainly composed of RBC exosomes) have been used as gene delivery vehicles to efficiently transport exogenous siRNA into monocytes and lymphocytes, leading to the selection of mitogen-activated protein kinase 1 sex gene silencing [66]. As RBCEVs normally do not activate the innate nor acquired immune system of the host, the repeated administration of RBCEVs is more applicable than viral vectors, which often suffer from immune reactions from the second dose. 

In general, there is a great deal of potential for RBCEVs to be used as carriers due to their superior biocompatibility, versality, and functionality. It is valuable to further investigate these natural vehicles for improved efficiency and more diverse uses.

#### 3.1.1. Liver Targeting

The liver is one of the most common accumulation sites of nanomedicine, and RBCEVs also share this property [67]. Compared with other organs, VivoTrack 680-labelled RBCEVs showed the highest accumulation in the liver after a mouse tail vein injection, which was related to C1q-dependent phagocytosis by macrophages [67]. In this way, drug-loaded RBCEVs could improve the therapeutic outcome in the treatment of liver-related diseases such as in situ liver cancer, liver failure, liver fibrosis, and a fatty liver. It has been reported that cationic amylopectin modified on the surface of EVs can enhance the precise targeting of the asialoglycoprotein receptor on hepatocytes, improving cell-specific delivery and the therapeutic effect of EVs [68]. Other modifications with RBCEVs are also possible to achieve similar results by increasing the delivery precision.

#### 3.1.2. Brain Targeting

The blood–brain barrier is one of the most difficult barriers for drugs/agents to cross in the body. Brain delivery is, therefore, still a major challenge. To solve this problem, various drug delivery systems, including EV-based systems, have been developed [69]. Various cells can produce EVs that can target the brain cells. For example, EVs from mesenchymal stem cells in different brain pathologies, including strokes, autism, Parkinson’s disease, and Alzheimer’s disease specifically targeted and accumulated in the brains of pathologically relevant murine models [70]. EVs secreted by monocytes and macrophages were shown to avoid entrapment in mononuclear phagocytes whilst being readily taken up by neuronal cells [71]. The brain cells themselves also secreted EVs to communicate between cells and to deliver bioactive molecules to recipient cells to modulate cell functions such as M2 microglia-derived EVs [72]. However, not all of these EVs efficiently passed the BBB without engineering, although they could target the brain cells. Compared with these EVs, RBCEVs can actively target the brain without surface modification, and are generally produced more easily [73,74]. It has been shown that RBCEVs without any modification move through the BBB based on the transferrin–TfR interaction and the formation of a transferrin dimer [73] (Figure 2). RBCEVs are α-syn-rich EVs and have been shown to favor a BBB crossing under inflammatory conditions provoked by the peripheral administration of lipopolysaccharide [74]. It has been reported that non-modified RBC exosomes carrying dopamine showed a 10-fold increase in dopamine in the brain.

The brain-targeting efficiency of RBCEVs may be further improved by reducing off-target accumulation such as the liver [67]. For instance, in order to reduce toxicity to non-target tissues, RBCEVs can be modified to have additional RVG-targeting capabilities [75]. 

#### 3.1.3. Tumor Targeting

Unmodified RBCEVs may naturally target tumors. It has been found that blood TfR^+^ exosomes, which are most likely generated by RBCs, can target tumors with their innate passive-targeting capabilities [76]. After loading with DOX, the tumor accumulation of DOX in tumor-bearing mice significantly increased [76]. Furthermore, the blood TfR+ exosomes altered the DOX biodistribution, resulting in reduced side effects and better tumor suppression compared with free DOX [76]. 

**Figure 3:**
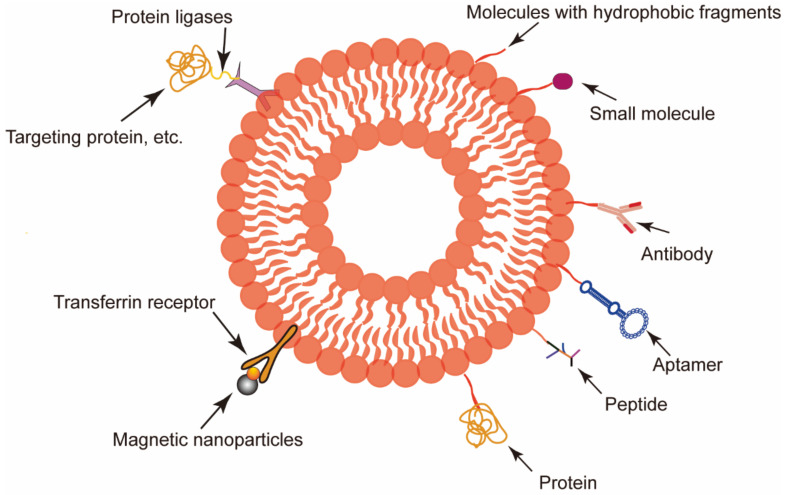
Surface modification method of red blood cell-derived extracellular vesicles (RBCEVs). RBCEVs were modified to obtain targeting ability by utilizing the proteins on the surface of RBCEVs and taking advantage of the properties of the cell membrane.

However, tumors occur in vastly different forms with highly diverse structures and characteristics. Thus, the natural tumor-targeting capability of RBCEVs is usually unsatisfactory for clinical applications [77]. Faced with a complex tumor microenvironment, additional engineering is often essential to create RBCEVs with a stronger tumor-targeting ability or a higher targeting specificity [78]. Unlike most other EVs, it is difficult to add target peptides or homing peptides to the surface of RBCEVs by transfection plasmids into their parent cells as mammalian mature erythrocytes do not have ribosomes [79,80]. Therefore, other strategies such as a membrane-anchoring molecule insertion or a peptide attachment can be applied (Figure 3).

Surface modifications using proteins such as CD47 and TfR have been well-covered by Thangaraju et al. [7,81]. By exploiting the characteristics of the cell membranes and adopting the principle of “similar compatibility”, targeting ligands were attached to the surface of the RBCEVs by linking the hydrophobic units with the targeting ligands. Covalently binding the peptide and cholesterol to the amine-functionalized AS1411 via the -COOH group allowed the cancer-targeting ligand T-AS1411 to bind to the RBCEV membrane, thus targeting nucleolin in breast cancer [82]. In addition to cholesterol, diacyl lipid tails also have hydrophobic interactions with cellular phospholipid layers. Zou et al. [83] developed an aptamer-equipped exosome platform for targeting cancer with chemotherapy drugs using diacyl liposome conjugates as functional ligands. In another study, the tumor-targeting ligand c(RGDyK) was introduced into the erythrocyte membrane surface through the covalent interaction of biotin and avidin by taking advantage of the hydrophobic interaction of DSPE with the cell membrane [84]. Similar modifications have also been applied to macrophage-derived exosomes, targeted by their surface-specific antibodies [85]. There are also other interesting methods to link peptides to the surface of RBCEVs such as the use of protein ligases (such as a sortase A heptamutant and an OaAEP1 Cys247Ala ligase) and “click chemistry” [86,87,88].

Substances other than biomolecules can also be used for surface modifications. In one study utilizing a transferrin receptor on the surface of RBCEVs, magnetic nanoparticles were used [89]. With an external magnetic field, RBCEVs modified with superparamagnetic nanoparticle clusters achieved tumor targeting [89]. Furthermore, after the surface modification of RBCEVs with magnetic molecules and L17E peptides, the engineered EVs loaded with DOX and miR-21i exhibited an enhanced tumor accumulation and improved endosomal escape, enabling the efficient and specific delivery of the encapsulated cargo to the tumor cells [90].

#### 3.1.4. Other Applications

RBCEVs can also be used in many other situations. As previously described, RBCEVs are closely linked to malaria. When antimalarial drugs (atovaquone and tafenoquine) were loaded into *Plasmodium falciparum*-infected RBCEVs (pRBCEVs), the treatment significantly improved the in vitro *P. falciparum* growth inhibition compared with the free drugs. This indicated that pRBCEVs can potentially increase the efficacy of several other small hydrophobic drugs used for the treatment of malaria [91]. 

Another interesting use of RBCEVs is to improve the delivery efficiency of other nanomedicines. As introduced above, nanomedicine (including EVs) normally accumulates in the liver regardless of the targeting strategy, thereby reducing the delivery efficiency to other sites. In order to improve the targeting efficacy to non-liver sites, the strategy of using nanoparticles to saturate the liver mononuclear phagocytosis system may be adopted [92]. It has been shown that injecting blank EVs before the administration of nanomedicine can reduce the nanomedicine level in the liver and improve the final therapeutic effect of drugs [93].

### 3.2. RBCEVs as a Therapeutic Target

RBCEVs transmit messages from the parent cells to different target cells throughout the body via a direct cell-to-cell contact or the passing of effector molecules. Several studies have suggested that RBCEVs may contribute to pathological processes [32,67,94]. Therefore, RBCEVs themselves may be good targets to abrogate the communication between cells at or adjacent to lesions, thereby limiting disease progression. However, the restriction of communication between RBCEVs and disease-related cells without affecting normal cellular signals remains challenging.

One possible approach is to target the underlying molecular mechanisms of the formation, release, and uptake of RBCEVs. The inhibition of ceramide synthesis during EV formation using a small molecule inhibitor of neutral sphingomyelinase or via the antihypertensive drug amiloride (which normally attenuates the circulation of endocytic vesicles) impaired the production of RBCEVs [95,96]. The inhibition of the RBCEV release by targeting GTPases such as RAB27A [97], RAB11 [98], or RAB35 [99] has also attracted research interest. Targeting intercellular adhesion molecule 1 (ICAM1) successfully prevented RBCEVs from reaching the uptake pathway [100]. Moreover, heparin sodium, cytochalasin D, and methyl-beta cyclodextrin were also found to inhibit the uptake of EVs [101]. These studies have shown the many possibilities of the RBCEV signaling blockade. However, these tools are still in the early stages of development and remain largely untested in disease models.

A better understanding of the formation, release, and uptake mechanisms of EVs is needed. It is also worth noting that many of these targets are also present in normal EVs; therefore, targeting these biomolecules may not only inhibit the functioning of the EVs that drive the disease progression, but also affect the EVs derived from normal cells. As the role of EVs secreted by healthy cells has not yet been fully explored, further investigations are required to verify the viability of these blocking approaches.

### 3.3. RBCEVs in Diagnosis and Prognosis

Reliable biomarkers can help doctors diagnose, evaluate, and predict the onset and progression of a disease. EVs are released by various types of cells under normal and pathological conditions, and can be found in a variety of body fluids. These EVs carry a variety of important molecules, providing a window into the altered cellular or tissue state. In this way, EVs from biological fluids become an attractive and minimally invasive method for a liquid biopsy [102]. For instance, EVs have been detected in amniotic fluid, suggesting the potential of EVs as biomarkers for prenatal diagnoses [103]; EVs in urine are attractive markers for genitourinary diseases [104], including prostate cancer. EVs may contain aquaporin 1 and aquaporin 2, which are useful markers of a renal ischemia/reperfusion injury and vasopressin action, respectively [105]. 

RBCEVs isolated from blood have been recognized as biomarkers of vascular damage and inflammation in a variety of cardiovascular diseases, including diabetes, preeclampsia, hypertension, and metabolic syndrome [106,107,108,109]. Recent studies have shown that RBCEVs may reflect pathophysiological changes in the brain instead of using cerebrospinal fluid [110]. This provides a new, less invasive approach for Alzheimer’s disease diagnosis and prognosis. The miRNAs contained in the RBCEVs obtained from blood have various pathophysiological characteristics of the parent cells and the genetic information in them can be used to analyze disease-related information [111]. It has been reported that cancer-specific miRNAs (miR-103, miR-191, and miR-195) in RBCEVs can be used to precisely differentiate healthy individuals from breast cancer patients [112]. The miR-451a level in RBCEVs may indicate the age of the host RBCs [113]. Even the mechanical properties of RBCEVs can be used for disease diagnoses. Patients with spherocytosis had RBCs that were too round and stiff; the EVs from these RBCs turned out to be softer [114].

In general, RBCEVs have shown promise in the diagnosis and prognosis of tumors as well as cardiovascular, liver, kidney, and other systemic diseases [115]. They have the advantages of a simple operation, being non-invasive, a low cost, low side effects, and a high compliance. More work is needed to expand the applications and standardize the process of RBCEV-based diagnosis/prognosis in order to fully exploit their potential.

## 4. Discussion

Although RBCEVs can offer many benefits, they also face several application challenges. For instance, the fast extraction of RBCEVs from blood in large quantities is still a challenge. The most commonly used method for RBCEV isolation is laborious ultra-high-speed differential centrifugation, which is difficult to scale up. Recently, a new extraction method based on acoustofluidics (i.e., the integration of acoustics and microfluidics) to directly produce RBCEVs from whole blood in a label-free and contact-free manner was published. This method, remarkably, saved extraction time whilst preserving the biological, biophysical, and structural integrity of the isolated EVs [116]. Gangadaran et al. [117] produced exosome mimetics from RBCs by one-step extrusion, which increased the yield by 130-fold and offered a possible solution for the mass production of EVs. Another problem is that the blood bank, as the source of RBCEVs, is usually stored for a certain period of time. Several studies have reported a significant increase in RBCEV concentration after storage at 4 °C [97,98]. RBCEVs generated after storage are thought to be associated with adverse effects and, potentially, immunosuppression in blood transfusion recipients [32,118,119]. It is, therefore, necessary to either improve the quality of RBCEVs generated from stored blood sources or slow the deterioration of the blood. These problems could be solved in other ways. For example, progress in stem cell research may make it possible to produce RBCs in vitro in large quantities.

As mentioned, RBCEVs carry many functional cargoes, either inside or on the surface. The detailed functions of these cargoes are yet to be clarified, and are important scientific issues. From another aspect, when loading other drugs/agents into the RBCEVs, these substances may be removed, at least partially. This may improve or diminish the efficiency of the RBCEV carriers. Therefore, the drug/agent loading technique and possible selective removing/adjustment of the innate cargoes of the RBCEVs are also important issues in the application of RBCEVs.

Finally, in clinical applications, RBCEVs could retain their activity under simple conditions for weeks or under −80 °C for months/years, thereby simplifying storage and transportation. However, there is still a demand to further elongate the storage time and simplify the storage conditions; hence, techniques such as freeze-drying need to be developed for EVs.

## 5. Conclusions

The generation and release of RBCEVs play an integral role in RBC maturation, aging, and disease. These links between RBCEVs and RBCs, as well as the multiple roles that RBCEVs play in human body, give RBCEVs a variety of potential medical uses. RBCEVs can naturally accumulate in the liver and pass the BBB, and the active targeting of RBCEVs to other sites can be achieved by surface modifications. They are versatile carriers of various different cargos. Therefore, RBCEVs could be used as excellent nanodelivery platforms for disease treatments. On the other hand, because of their complex roles under physiological conditions, they may also be treated as therapeutic targets or used for disease diagnoses and prognoses. These vesicles clearly have potential and may open new possibilities for precise medicine. However, many aspects of RBCEVs are still unclear such as how RBCEVs interact with other tissues and organs [120]; there are still challenges for their clinical translation. Therefore, research based on different disciplines is needed to realize the full potential of RBCEVs.
